# Non-Immersive Virtual Reality to Improve Balance and Reduce Risk of Falls in People Diagnosed with Parkinson’s Disease: A Systematic Review

**DOI:** 10.3390/brainsci11111435

**Published:** 2021-10-28

**Authors:** Héctor García-López, Esteban Obrero-Gaitán, Adelaida María Castro-Sánchez, Inmaculada Carmen Lara-Palomo, Francisco Antonio Nieto-Escamez, Irene Cortés-Pérez

**Affiliations:** 1 Department of Nursing, Physical Therapy and Medicine, University of Almeria, Road Sacramento s/n, 04120 Almeria, Spain; hector.garcia@ual.es (H.G.-L.); adelaid@ual.es (A.M.C.-S.); inma.lara.palomo@gmail.com (I.C.L.-P.); 2 Department of Health Sciences, University of Jaen, Paraje Las Lagunillas s/n, 23071 Jaen, Spain; eobrero@ujaen.es (E.O.-G.); icp00011@red.ujaen.es (I.C.-P.); 3 Department of Psychology, University of Almeria, Ctra. Sacramento s/n, 04120 Almeria, Spain; 4 Center for Neuropsychological Assessment and Rehabilitation (CERNEP), Ctra. Sacramento s/n, 04120 Almeria, Spain; 5 Granada Northeast Health District, Andalusian Health Service, Street San Miguel 2, 18500 Guadix, Spain

**Keywords:** rehabilitation, Parkinson’s disease, VR, virtual reality, non-immersive, risk of falls, balance

## Abstract

(1) Objective: To evaluate the effectiveness of non-immersive virtual reality in reducing falls and improving balance in patients diagnosed with Parkinson’s disease. (2) Methods: The following databases were searched: PUBMED, PEDro, Scielo, CINAHL, Web of Science, Dialnet, Scopus and MEDLINE. These databases were searched for randomized controlled trials published using relevant keywords in various combinations. The methodological quality of the articles was evaluated using the PEDro scale. (3) Results: A total of 10 studies with a total of 537 subjects, 58.7% of which (n = 315) were men, have been included in the review. The age of the participants in these studies ranged between 55 and 80 years. Each session lasted between 30 and 75 min, and the interventions lasted between 5 and 12 weeks. These studies showed that non-immersive virtual reality is effective in reducing the number of falls and improving both static and dynamic balance in patients diagnosed with Parkinson’s disease. Results after non-immersive virtual reality intervention showed an improvement in balance and a decrease in the number and the risk of falls. However, no significant differences were found between the intervention groups and the control groups for all the included studies regarding balance. (4) Conclusions: There is evidence that non-immersive virtual reality can improve balance and reduce the risk and number of falls, being therefore beneficial for people diagnosed with Parkinson’s disease.

## 1. Introduction

Parkinson’s disease (PD) is a chronic, progressive condition characterized by the loss of dopaminergic neurons located in the substantia nigra of the *Pars Compacta* (SNpc) of the midbrain, which eventually leads to depletion of the neurotransmitter dopamine in the basal ganglia [1,2]. It is considered the second most common neurodegenerative disorder, and affects 2%–3% of the population over 65 years of age [3,4]. The quality of life of patients with PD decreases considerably due to changes in both motor and non-motor functions. The resulting functional disability places a considerable physical and mental burden on family members and caregivers [5]. The clinical manifestations of PD are characterized by slow movements, resting tremor and rigidity, together with non-motor manifestations. The most common feature of PD is bradykinesia, a progressive slowness in carrying out movements, including difficulties for planning, initiating and executing tasks that require simultaneous and sequential movements, such as ambulation [6].

This leads to postural instability typical of PD, and largely contributes to the high risk of falls in PD patients [7]. Losing their ability to keep their balance while standing also undermines patients’ quality of life and their functionality, and considerably increases the risk of falls [8]. As the disease progresses, PD patients lose postural stability, which in turn causes gait disorders and limitations in their basic, instrumental and advanced activities of daily living [9]. Although motor abnormalities such as resting tremor may improve with medication, other symptoms such as postural instability while standing do not respond to medication and require alternative therapeutic approaches [10].

Currently, the most widely used pharmacological treatment to manage the motor symptoms associated with PD is dopamine replacement and/or dopamine agonist therapy [11]. Treatment with L-Dopa improves patient quality of life by alleviating the motor symptoms associated with dopamine depletion [12]. However, since neuronal death continues, L-dopa must be successively up-dosed, and it usually loses its effectiveness after several years of chronic use. Chronic (5–10 years) treatment with L-Dopa also causes certain side effects, such as dyskinesia [13], hence the importance of introducing new therapeutic interventions that can diminish the impact of dyskinesia in PD [14].

Various therapies are being used as complement to pharmacological treatment in PD, such as: physical activity [15], deep brain stimulation (DBS) [16], transcranial magnetic stimulation (TMS) [17], cell replacement [18] or virtual reality therapy [19]. The latter is one of the latest techniques in the field of neurorehabilitation, ageing and disability [20].

Virtual reality (VR) is defined as a 3-dimensional (3D) computer generated environment in which the user is able to see, hear or manipulate the contents of such artificial environment [21]. The 3D environments can vary depending on their level of immersion. VR can be classified as immersive, semi-immersive and non-immersive [22]. The last two have been named “non-immersive” due to the lack of fully multisensory simulation, and the user still perceives some information from the real world [23]. Thus, in non-immersive VR systems (NIVR), subjects interact with a scenario displayed on a screen, but do not become completely immersed because they are able to perceive the real world together with the digital images. Most of these systems can use a joystick to interact with a PC or tablet [24]. Semi-immersive VR takes the subjects to a partially immersive scenario displayed on a screen, and frequently they able to interact with the digital scene through body movements. The disadvantage of this type of simulations is that users are susceptible to environmental distractions [25]. Some examples of devices used in semi-immersive VR are: Holobench, IMAX, DOMES and Inmersadesk [26].

Nevertheless, non-immersive systems traditionally have offered a number of advantages over immersive VR, such as low cost and user-friendliness, since they permit an individual to maintain contact with the real world [27].

One of the main advantages of VR systems is that they allow to develop different intervention protocols in which the therapist can change the content, duration, intensity and feedback. It is even possible to use VR technology in combination with other applications, such as brain-computer interface (BCI) technology that make possible to control avatars or objects in video games [28]. This training model is being used to promote neural reorganization and neuroplasticity, which is key during recovery from various neurological disorders, such as stroke, multiple sclerosis or infantile cerebral palsy (PCI), among others, and to improve balance and risk of falls [29].

The aim of this systematic review is to determine whether NIVR can be an effective complement to more conventional neurorehabilitation treatments in terms of improving postural stability while reducing the risk of falls in patients with PD.

The low cost and user-friendliness of non-immersive VR systems could result in a useful, and readily available tool for healthcare professionals in charge of rehabilitation of patients diagnosed with PD.

## 2. Materials and Methods

### 2.1. Study Design

This systematic review has been performed following the recommendations of the Preferred Reporting Items for Systematic Reviews and Meta-Analysis (PRISMA) (Moher, 2009) [30] and the Cochrane Handbook for Systematic Reviews of Interventions (Cumpston, 2019) [31]. The methodology of the review was registered in the International Prospective Register of Systematic Reviews (PROSPERO), under the following number: CRD42021266966 (11 August 2021). Available from: https://www.crd.york.ac.uk/prospero/display_record.php?ID=CRD42021266966.

### 2.2. Source Data and Search Strategy

We performed a literature search in PubMed Medline, PEDro (Physiotherapy Evidence Database), SciELO, CINAHL Complete, Web of Science, Dialnet, and Scopus between May 2021 and August 2021. We also searched the references of full text articles together with the grey literature (conference abstracts, expert papers and clinical practice guidelines) for studies published until the moment of the search. The Cochrane Collaboration PICOS strategy was used to formulate the research question [32]: Is non-immersive virtual reality an effective strategy for improving balance and reducing the number of falls in Parkinson’s patients?, as shown in Table 1.

On this basis, we created a search strategy using Medline Medical Headings Subjects (MeSH) keywords, such as: “virtual reality”, “virtual reality exposure therapy”, “parkinson disease”, “postural balance” and “accidental falls” and synonyms (entry terms). We only reviewed those articles we had access to the full text. Table 2 shows the search strategy used for each database.

### 2.3. Study Design

We conducted a systematic review of the scientific literature by searching databases for published studies on the effectiveness of NIVR in preventing falls and improving balance in patients diagnosed with PD. This was followed by a critical analysis of the scientific literature retrieved from the literature search.

### 2.4. Study Screening: Inclusion and Exclusion Criteria

Three researchers performed the identification phase independently (F.A.N-E., I.C.L.-P, I.C.-P.). All studies selected by at least one of the investigators on the basis of the title and abstract were included for the final screening. Then, all the selected records were analyzed by two of these researchers. If consensus was not reached, the decision was made by a third researcher (A.M.C.-S.).

Studies included in the review had to meet the following inclusion criteria: (1) randomized clinical trial (RCT) or RCT pilot; (2) in which the effect of RVNI was analyzed; (3) compared to other interventions or simple observation; (4) on balance or risk of falls; (5) in Parkinson’s patients; and (6) RCTs with a methodological quality >4 on the PEDro scale. Exclusion criteria were: (1) studies other than RCTs; (2) studies in which the sample included a range of neurological pathologies apart from Parkinson’s and did not present their results disaggregated by pathology (3) single group studies.

### 2.5. Data Extraction

Two investigators (H.G.-L., E.O.-G.) extracted the data from the included studies, and discrepancies were resolved by consensus. Data were collected on the general characteristics of the study (authorship, year of publication, country and type of study), the characteristics of the sample (number of groups, participants per group and age of participants), the characteristics of the intervention (type of NIVR system, number of weeks, number of sessions per week, duration of each session and evaluation schedule).

### 2.6. Outcomes

The main outcome variables analyzed in this review were balance and risk of falls. Balance was analyzed using the Berg Balance Scale (BBS), the Activities-specific Balance and Confidence (ABC) scale, the Tinetti scale, and dynamic posturography performed using the balance master system (NeuroCom International Inc, Clackamas, OR). The risk of falls and balance confidence were analyzed using the Timed Up and Go Test (TUG) and the Functional Reach Test (FRT). These instruments have been used for such goal in previous studies [33,34]. The number of falls was also measured through self-report instruments, see Table 3.

### 2.7. Risk of Bias Assessment

The PEDro scale [35,36]—a checklist of 11 yes-or-no questions—was used to assess the methodological quality and risk of bias of the articles selected for the systematic review. The final score is the sum of answers 2 to 11, giving a score of between 0 and 10. A study is “excellent” if it has a score of 9–10; “good quality” if it has a score of 6 to 8 points; “moderate quality” if it scores between 4 and 5 points, and “low quality” if it scores less than 3. The eligibility criteria are not used to calculate the final score.

## 3. Results

### 3.1. Search Results

**Figure 1:**
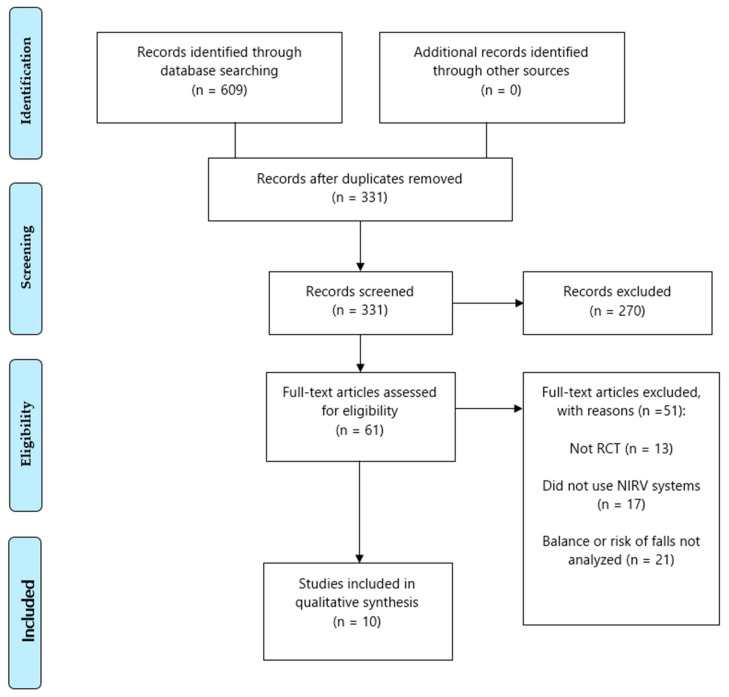
Eligibility and data synthesis: PRISMA flow diagram.

The initial search identified 609 potential articles (PubMed Medline, 130; Web of Science, 266; PEDro, 33; SCOPUS, 152; CINAHL, 18; SciELO, 7; Dialnet, 3), of which 278 were duplicates and therefore excluded, leaving 331 articles to review in full text. After reviewing the abstract, 270 articles were excluded, leaving 61 articles to be evaluated in full text due to their eligibility; 51 articles were excluded for the following reasons: different to RCT (13); does not use NIVR systems (17); and balance or risk of falls are not analyzed (21). Therefore, 10 studies were included in the systematic review. Figure 1 shows the PRISMA flow diagram with the different phases of the review [30] (eligibility and data synthesis. PRISMA flow diagram).

### 3.2. Characteristics of the Included Studies

A total of 537 participants were included in the 10 studies reviewed. The mean age of participants was 69 years; and there were 51 dropouts. Participants dropped out or were withdrawn for the following reasons: change in treatment; loss of interest or low motivation; personal reasons and adverse events; medical reasons; difficulty in travelling to the study site; and non-compliance with the treatment protocol. The mean number of participants in the intervention group after randomization was 28 subjects diagnosed with PD, with a range of between 10 and 66 subjects; three studies had more than 30 subjects in the intervention group [37,41,42].

The NIVR rehabilitation protocols differed in terms of the device used, the time per session and frequency of treatment, and the duration of the intervention. The devices used were Nintendo Wii Fit [39,40,41,43,45,46], modified Microsoft Kinect connected to a large screen [37,38,42] and a custom-created non-immersive VR system consisting of a 22-inch touch screen and a balance board [44]. In one study, the frequency of treatment differed between the groups (twice a week for controls and three times a week for the experimental group) [43]; however, in the remaining nine studies [37,38,39,40,41,42,44,45,46] the average frequency of treatment was three times per week (range three to five times per week). The average session time using NIVR was 53.5 min (range 30–75 min). Table 4 summarizes interventions characteristics of the revised studies.

In the experimental groups, NIVR consisted of a combination of treadmill training [37,38,42,46], conventional exercise programs [39], neurodevelopmental exercises [45] and functional electrical stimulation [45]. Treatment was carried out at home [41,44], and using NIVR alone [40,43]. In two studies, the NIVR program was applied at home [41,44], and in the remaining studies it took place in a clinical and experimental setting [37,38,39,40,42,43,45,46]. Several outcome measures were used to assess the efficacy of NIVR rehabilitation in the management of patients with PD. In five studies, the outcome measure was the number of falls [37,38,41,42,46]. There were significant differences in the number of patient-reported falls between the intervention and control groups in three of these studies [38,42,46].

### 3.3. Methodological Quality of Included Studies

The 10 studies [37,38,39,40,41,42,43,44,45,46] included in this systematic review were assessed for their methodological quality and risk of bias using the PEDro scale [35,36], as described in Table 5. The methodological quality of the included studies ranged from 4 to 8 on a scale of 11; criterion 1 of illegibility was not considered for the total score. The mean score was 6.1, which shows good overall methodological quality. No article showed low methodological quality, three studies were of moderate methodological quality [37,38,45], seven were moderate to high [39,40,41,42,43,44,46], and none was rated excellent.

### 3.4. Results of the Included Studies

#### 3.4.1. Balance

Balance was analyzed using different scales in seven of the 10 studies [39,40,41,43,44,45,46]. The BBS was used to assess the static and dynamic balance skills of patients diagnosed with PD [39,40,41,43,44,45], the ABC scale was used to assess balance confidence in specific activities [41], one study used the Tinetti scale to assess balance and gait [43], and finally, one study tested sensory organization and dynamic balance using a dynamic posturography system called the Balance Master (NeuroCom International Inc., Clackamas, OR, USA) [46]. Three of the studies analyzed found no significant differences in balance between the intervention groups and the control group. [39,43,44].

Yang et al. [44] reported an improvement in balance in the group performing home NIVR compared to the group performing a traditional physical therapy program. This is consistent with the findings of Gandolfi et al., [41] who observed significantly greater improvement in the home NIVR group versus the group receiving sensory integration balance training.

After six weeks of treatment, Lee et al. [45] found significant improvement in balance in the group undergoing NIVR in combination with neurodevelopment therapy and functional electrical stimulation. In contrast, the control group that underwent combined neurodevelopmental therapy and functional electrical stimulation showed no statistically significant improvements.

Liao et al. [46] performed a study with three intervention groups. The experimental groups performed a conventional exercise program combined with treadmill training or NIVR combined with treadmill training, and were compared to a control group who only received a fall prevention educational program. The authors found significant differences between the experimental groups and the control group in terms of dynamic balance and sensory organization (evaluated using a clinical posturology instrument).

#### 3.4.2. Risk of Falls

Pelosin et al., [38] similar to Mirelman et al., [42] reported an improvement in the number of falls in the experimental group after NIVR treatment combined with treadmill training compared to the treadmill-only group.

In a study with three intervention groups, Liao et al. [46] observed an improvement in the number of falls in the group performing NIVR in combination with treadmill training compared with the control group, which only received fall prevention education. However, they found no difference between the group performing NIVR combined with treadmill training and the group performing conventional exercises plus treadmill training.

Five studies [39,40,43,44,46] evaluated the risk of falls using the TUG test [39,40,44,46] and the functional range of motion test [43]. In three of these studies, the authors observed no significant differences in the risk of falls between the intervention and control groups [39,43,44].

Feng et al. [40] found significant differences between the intervention group performing NIVR compared to a traditional physiotherapy program.

Liao et al. [46] observed that the risk of falls in the group performing NIVR combined with treadmill training improved with respect to the control group receiving fall prevention education.

## 4. Discussion

Parkinson’s disease is a movement disorder characterized by disordered communication between the visual, vestibular, and proprioceptive systems. Posture deficits are also frequently observed in these patients. Postural stability is known to depend on good coordination between the visual, vestibular and proprioceptive systems [47].

Since PD is a chronic progressive disease, rehabilitation is a long process that requires patient cooperation. One of the advantages of VR in the rehabilitation of PD patients is that it maintains patient motivation, resulting in a useful tool for long-term treatments and to maintain gait and postural performance in PD patients.

The present work has analyzed 10 CRT studies evaluating the effectiveness of NIVR as intervention strategy for risk of falls and balance rehabilitation in PD patients. The quality of these studies has been positively rated according to PEDro and comprised a total sample of 537 PD patients. Additionally, less than 10% of participants dropped out in the original studies, which can be considered a positive result regarding the adherence to the treatment. Despite the different approaches to NIVR described in the revised studies, this technique was found to be effective in improving static and dynamic balance in patients with PD, and for reducing the rate and risk of falls. The variety of approaches reported in the studies reviewed also illustrates the nature and diversity of NIVR procedures used in the treatment of this population and, therefore, supports the clinical validity of our findings.

NIVR has been shown to be more effective than conventional physical therapy for balance and gait rehabilitation in PD patients [40]. The authors referred that visual feedback from virtual activities is a relevant factor for PD patients during the rehabilitation process. Moreover, it has also been observed that NIVR combined with other therapeutic tools, such as treadmill training [37,38,42,46], conventional exercise [39] or functional electrical stimulation along with neurodevelopmental treatment [45], significantly improved balance and reduced falls in PD patients. In the same line, patients following NIVR programs at home have also shown an improvement in static and dynamic postural control, balance and walking function [41,44], showing that home-based VR might be a viable option for PD balance training.

Of the 10 studies included in this review, only Negrini et al. [43] used NIVR in both treatment groups, with 10 sessions in the control group and 15 in the experimental groups. These authors found significant differences in balance and fall rates between groups, but no significant differences in Tinetti test results.

The mean duration of NIVR treatment was 6.8 weeks (range 5 to 12 weeks), with between two to five sessions per week [37,38,39,40,41,42,43,44,45,46]. PD patients received an average of 25 NIVR sessions [37,38,39,40,41,42,43,44,45,46]. However, this treatment intensity places a considerable burden on financial and human resources.

The physiotherapy evidence database PEDro scores for 7 of the 10 articles included ranged from 6 to 8, indicating that they are of moderate to high methodological quality [39,40,41,42,43,44,46]; the remaining three studies were of moderate methodological quality [37,38,45].

Some authors have found that VR helps PD patients adjust segmental trunk alignment [40], while others have reported that VR games can also improve the patient’s standing stability by improving organization and integration within the vestibular system [48]. Thus, VR games provide dynamic and static posture control activities that help PD patients improve control of their trunk and center of gravity, which in turn allows them to adjust their segmental trunk alignment. Visual feedback in VR games, therefore, allows patients to sense their own position and direction of movement in space based on visual tracking and to coordinate their body position. Some authors claims that multisensory perceptual feedback in VR rehabilitation promotes neural networking in cortical and subcortical areas of the brain [49]. Neuroimaging studies have shown that virtual motion can activate motion-related areas in the brain, a finding that supports its role in rewiring and reorganizing the affected brain circuits [50]. Thus, VR combined with immediate multisensory feedback facilitates task repetition and drives neural changes in the corresponding cortex. This reduces the fear of falling, and transfers this confidence into the real world through motor learning [51]. Amirthalingam et al. [52] recently suggested that task repetition using VR increases neural plasticity in both post-stroke patients and patients diagnosed with Parkinson’s disease

It is imperative to mention that studies included in this review present several weaknesses and methodological limitations. The first concern is the reduced sample size, ranging between 20 and 130 patients. In addition, the method used to determine the sample size was not reported in some studies, thus limiting the external validity of their findings. In all but one of the studies, the treating therapists were not blinded [43]. In two studies, there was no confirmation that assessors measuring at least one of the key outcomes had been blinded [37,45], and there was no mention of patient blinding in any of the studies [37,38,39,40,41,42,43,44,45,46].

Although these shortcomings may have increased the risk of bias in these studies, it may not be feasible to blind participants or therapists in a clinical trial using this treatment tool. Furthermore, some important outcomes, such as balance or the number of falls, were not evaluated in all the studies reviewed.

We believe that more research is needed to evaluate the effectiveness of NIVR on these treatment outcomes in patients with PD. This systematic review also has certain limitations. We only included studies published in English; therefore, we cannot be sure that relevant scientific literature published in different languages was not overlooked. Furthermore, as we only included studies we had full access, relevant information about the effectiveness of NIVR in PD patients may have been overlooked. To the best of our knowledge, this is the first review of scientific evidence on the effectiveness of NIVR as a tool for improving balance and reducing the number and risk of falls in patients diagnosed with EP. Professionals in the field of neurorehabilitation should be aware of the outcomes achieved with NIVR devices in the treatment of PD, since evidence has shown that it can be a valuable tool in the context of rehabilitation programs. Thus, NIVR alone has shown to be more efficient than traditional intervention programs [40]. Additionally, NIVR increases the effectiveness of other therapies such as treadmill training [37,38,42,46], exercises programs [39,44] or functional electrical stimulation [45] producing a larger effect on the risk of falls and balance compared to their application alone.

## 5. Conclusions

The studies analyzed show that NIVR-based therapy programs lasting between 6 and 12 weeks can significantly reduce the number of falls in PD patients. Although the mixed results reported in these studies show that there is no clear evidence about the superiority of NIVR over other therapies, such as exercise programs or conventional physiotherapy.

NIVR combined with treadmill training has proven more effective than NIVR alone.

Home NIVR rehabilitation programs have shown to be effective in preventing falls and improving balance in PD patients.

Nevertheless, future studies about NIVR programs should be conducted in larger and more homogeneous samples. Moreover, studying patients following NIVR programs in isolation would help determine the effectiveness of this therapeutic approach. In the same line, the most efficient intervention protocol using NIVR should be defined, also comparing the effectiveness of different NIVR tools. Moreover, it will be fundamental that control protocols are carried out in a more homogeneous way and defined with more detail.

## Figures and Tables

**Table 1:** PICOS: Participants, Interventions, Comparisons, Outcomes and Study design.

Participants	Interventions	Comparisons	Outcomes	Study Design
Patients with Parkinson’s	NIVR	Fall prevention education, treadmill, conventional exercise, and sensory integration balance training	Index of falls, balance, functional mobility and motor status	Randomized clinical trials

Notes: PD = Parkinson’s Disease; RVNI = non-immersive virtual reality; VR = virtual reality.

**Table 2:** Search strategies used in each database.

Database	Search strategy
PubMed Medline	(parkinson disease[mh] OR parkinson disease[tiab] OR parkinson’s disease[tiab] OR “parkinson”[tiab]) AND (virtual reality[mh] OR virtual reality[tiab] OR virtual reality exposure therapy[mh] OR “non-immersive virtual reality”[tiab] OR “Nintendo”[tiab] OR “Xbox” [tiab] OR videogam *[tiab] OR exergame *[tiab]) AND (postural balance[mh] OR postural balance[tiab] OR “balance”[tiab] OR postural control[tiab] OR accidental falls[mh] OR accidental falls[tiab] OR fall *[tiab] OR risk of fall *[tiab])
PEDro	Parkinson * virtual reality
Web of Science	TS = (Parkinson * AND (videogame * OR exergame * OR virtual reality) AND (balance or fall *))
SCOPUS	(TITLE-ABS-KEY (parkinson OR “Parkinson’s disease”)) AND (TITLE-ABS-KEY (“virtual reality” OR “exergames”)) AND (TITLE-ABS-KEY (“balance” OR “fall”))
CINAHL	(MH “Parkinson Disease”) AND ((MM “Virtual Reality Exposure Therapy”) OR (MM “Virtual Reality”) OR (MM “Exergames”)) AND ((MM “Balance, Postural”) OR (MM “Balance Training, Physical”) OR (MM “Accidental Falls”))
DIALNET	Parkinson * AND (“virtual reality” OR exergame *) AND (balance OR fall *)
SciELO	Parkinson * AND (“virtual reality” OR exergame *) AND (balance OR fall *)

**Table 3:** Characteristics of the studies included in the systematic review.

Study	Participants (N)	Age (years)	Design	Evaluation	Outcomes	Measuring Instrument	Results
Del Din et al. (2020) [37]	128	71.68 ± 6.4	CG = 62 EG = 66	T0 = Baseline T1 = 6 wk	Number of falls	FRA	The FRA index decreased significantly in the CG and EG (*p* ≤ 0.035).
Pelosin et al. (2020) [38]	24	71.9 ± 4.1	CG = 14 EG = 10	T0 = Baseline T1 = 6 w kT2 = 12 wk	Number of falls	Schedule	The EG and CG showed a significant time training interaction (F 1.33 = 7.39, *p* = 0.012). EG = TM + VR reduced the number of falls (*p* < 0.001) with respect to CG = TM.
Santos et al. (2019) [39]	45	64.3 ± 8.5	CG = 15 EG1 = 15 EG2 = 15	T0 = Baseline T1 = 8 wk	Balance Risk of falls	BBS TUG	No statistically significant differences between GG, EG1 and EG2 with respect to BBS (*p* = 0.968) and TUG (*p* = 0.824).
							Significant differences found in pre and post intervention analyses of all outcomes.
							The effect size was larger for EG2 = NW + CE in all functional tests.
Feng et al. (2019) [40]	28	66.93 ± 4.64 67.47 ± 4.79	CG = 14 EG = 14	T0 = Baseline T1 = 12 wk	Balance Risk of falls	BBS TUG	After Tx, BBS and TUG scores improved significantly in both groups (*p* < 0.005). The EG = VR showed improved performance compared to the CG = CP on BBS, TUG and Unified Parkinson’s Disease Rating Scale (*p* < 0.005).
Gandolfi et al. (2017) [41]	76	69.84 ± 9.41 67.45 ± 7.18	CG = 38 EG = 38	T0 = Baseline T1 = 7 wk T2 = 11 wk	Balance Balance confidence activities Number of falls	BBS ABC Self-reported	There were significant differences between the groups, with the EG = home VR showing improvement in the BBS (*p* = 0.04).
							No significant differences between the groups for ABC and number of falls. Significant pre/post-test differences in EG = home VR with respect to the number of falls (*p* = 0.034).
Mirelman et al. (2016) [42]	130	73 ± 5 74 ± 5	CG = 64 EG = 66	T0 = Baseline T1 = 6 wk T2 = 30 wk	Number of falls	Incidence	The number of falls was lower in the EG = TM + VR than in the CG = TM in patients diagnosed with Parkinson’s (*p* = 0.001).
Negrini et al. (2016) [43]	27	67 ± 9 66 ± 8	CG = 11 EG = 16	T0 = Baseline T1 = 5 wk T2 = 9 wk	Balance Risk of falls	BBS TT FRA	The post hoc analysis showed significant differences between groups in the pre-test, post-test and follow-up (*p* < 0.02) on BBS and FRA, but no significant difference between the pre-test and follow-up in the Tinetti test (*p* = 0.2) in the EG.
							No significant differences between the intervention groups (*p*> 0.005).
							The effect size was large in BBS (d = 0.9); moderate in TT (d = 0.4) and small in FRA (d < 0.2) after the intervention.
Yang et al. (2016) [44]	23	72.5 ± 8.4 75.4 ± 6.3	CG = 12 EG = 11	T0 = Baseline T1 = 6 wk T2 = 8 wk	Balance Risk of falls	BBS TUG	Both groups obtained better results in relation to BBS and TUG after the intervention and at 8 weeks of follow-up (*p* < 0.001).
							No significant differences between the groups after the test and at 8 weeks of follow-up.
Lee et al. (2015) [45]	20	70.1 ± 3.3 68.4 ± 2.9	CG = 10 EG = 10	T0 = Baseline T1 = 6 wk	Balance	BBS	After 6 wk of Tx, BBS improved significantly in the EG (46.0 ± 1.3 to 48.1 ± 3.0; *p* < 0.05), but showed no significant improvement in the CG (45.0 ± 1.3 to 45.4 ± 1.5; *p* > 0.05).
Liao et al. (2015) [46]	36	64.6 ± 8.6 65.1 ± 6.7 67.3 ± 7.1	CG = 12 EG1 = 12 EG2 = 12	T0 = Baseline T1 = 6 wk T2 = 10 wk	Dynamic balance Sensory organization Risk of fallsNumber of falls	MV/SOT TUG FES-I	EG1 and EG2 showed significant improvements in MV/SOT compared to the CG after treatment and at 1 month of follow-up (*p* < 0.001).
							EG1 and EG2 showed significant improvements compared to the CG relative to follow-up (*p* < 0.001).
							No significant differences between EG1 and EG2 relative to FES-I.
							EG2 showed significant improvements in SOT, TUG, FES-I with respect to CG.

ABC = Activities-specific Balance Confidence Scale; BBS = Berg balance scale; CE = Conventional exercise; CG = Control group; CP = Conventional physiotherapy; EG = Experimental group; FES = Functional electrical stimulation; FES-I = Falls Efficacy Scale; FPE = Fall prevention education; FRA = fall rates relative to activity exposure index; HE = Healthy elderly patients; HT = Home training; IF = Idiopathic falls; MCI = Mild cognitive impairment; MV = Dynamic balance test; NDT = Neurodevelopmental treatment; NW = Nintendo Wii Fit; OA = Osteoarthritis; TM = Treadmill; TT = Tinetti Test; TUG = Timed Up and Go; Tx = Treatment; SIBT = Sensory Integration Balance Training; SOT = Sensory organization test; VR = virtual reality; WK = Weeks.

**Table 4:** Characteristics of interventions used in the included studies.

Study	Intervention	Type of NIVR	Time per Session	Frequency	Duration of Treatment
Del Din et al. (2020) [37]	CG = TM EG = TM + NIVR	Large screen Modified Microsoft Kinect	40 min	3/wk	6 wk
Pelosin et al. (2020) [38]	CG = TM EG = TM + NIVR	Large screen Modified Microsoft Kinect	45 min	3/wk	6 wk
Santos et al. (2019) [39]	CG = CE EG1 = NIVR EG2 = NIVR + CE	Nintendo Wii Fit	50 min	2/wk	8 wk
Feng et al. (2019) [40]	CG = CP EG = NIVR	Nintendo Wii Fit	45 min	5/wk	12 wk
Gandolfi et al. (2017) [41]	CG = clinical SIBT EG = home NIVR	Nintendo Wii Fit	50 min	3/wk	7 wk
Mirelman et al. (2016) [42]	CG = TM EG = TM + NIVR	Large screen Modified Microsoft Kinect	45 min	3/wk	6 wk
Negrini et al. (2016) [43]	CG = NIVR 10 ss EG = NIVR 15 ss	Nintendo Wii Fit	30 min	CG = 2/wk EG = 3/wk	5 wk
Yang et al. (2016) [44]	CG = CE	Touch screen	50 min	2/wk	6 wk
	EG = home NIVR	Virtual balance training system			
Lee et al. (2015) [45]	CG = NDT + FES EG = NDT + FES + NIVR	Nintendo Wii Fit	45 min 75 min	5/wk	6 wk
Liao et al. (2015) [46]	CG = FPE	Nintendo Wii Fit	60 min	2/wk	6 wk
	EG1 = CE + TM				
	EG2 = NIVR + TM				

ABC = Activities-specific Balance Confidence Scale; BBS = Berg balance scale; CE = Conventional exercise; CG = Control group; CP = Conventional physiotherapy; EG = Experimental group; FES = Functional electrical stimulation; FES-I = Falls Efficacy Scale; FPE = Fall prevention education; FRA = fall rates relative to activity exposure index; HE = Healthy elderly patients; HT = Home training; IF = Idiopathic falls; MCI = Mild cognitive impairment; MV = Dynamic balance test; NDT = Neurodevelopmental treatment; NW = Nintendo Wii Fit; OA = Osteoarthritis; TM = Treadmill; TT = Tinetti Test; TUG = Timed Up and Go; Tx = Treatment; SIBT = Sensory Integration Balance Training; SOT = Sensory organization test; VR = virtual reality; WK = Weeks.

**Table 5:** Assessment of methodological quality and risk of bias on the PEDro scale [35,36].

Study	Criterion											Total Score
	1	2	3	4	5	6	7	8	9	10	11	
Del Din et al. (2020) [37]	NO	YES	NO	YES	NO	NO	NO	NO	YES	YES	YES	5
Pelosin et al. (2020) [38]	YES	YES	YES	NO	NO	NO	YES	NO	NO	YES	NO	4
Santos et al. (2019) [39]	YES	YES	NO	YES	NO	NO	YES	YES	YES	YES	YES	7
Feng et al. (2019) [40]	YES	YES	NO	YES	NO	NO	YES	YES	YES	YES	YES	7
Gandolfi et al. (2017) [41]	YES	YES	NO	YES	NO	NO	YES	YES	NO	YES	YES	6
Mirelman et al. (2016) [42]	YES	YES	YES	YES	NO	NO	YES	YES	YES	YES	YES	8
Negrini et al. (2016) [43]	YES	NO	NO	NO	NO	YES	YES	YES	YES	YES	YES	6
Yang et al. (2016) [44]	YES	YES	NO	YES	NO	NO	YES	YES	YES	YES	YES	7
Lee et al. (2015) [45]	NO	YES	NO	YES	NO	NO	NO	NO	NO	YES	YES	4
Liao et al. (2015) [46]	YES	YES	YES	YES	NO	NO	YES	YES	NO	YES	YES	7

Data extracted from PEDro database. Criteria: 1, Eligibility criteria were specified (not used for score); 2, Subjects were randomly allocated to groups; 3, Allocation was concealed; 4, Groups were similar at baseline regarding the most important prognostic indicators; 5, There was blinding of all subjects; 6, There was blinding of all therapists who administered the therapy; 7, There was blinding of all assessors who measured at least one key outcome; 8, Measures of at least one key outcome were obtained from more than 85% of the subjects initially allocated to groups; 9, All subjects for whom outcome measures were available received the treatment or control condition as allocated or, where this was not the case, data for at least one key outcome was analyzed by ‘intention-to-treat’; 10, The results of between-group statistical comparisons were reported for at least one key outcome; 11, The study provides both point measures and measures of variability for at least one key outcome).Yes criteria met; No: criteria not met.

## Data Availability

All available data can be obtained by contacting the corresponding author.
